# High-Volume, High-Acuity, and High-Impact Learning: Tips and Tricks for Infectious Diseases Training Programs

**DOI:** 10.1093/ofid/ofae016

**Published:** 2024-01-08

**Authors:** Vera P Luther, Molly L Paras, Sara Schultz, Mariam Aziz, Gayle Balba, Todd P McCarty, Raymund R Razonable, Rebecca Reece, Rachel Shnekendorf, Vidya Sundareshan, Lisa M Chirch, the Infectious Diseases Society of America Infectious Diseases Training Program Directors Committee and Working Group, Emily Abdoler, Kartikey Acharya, Marwan. M Azar, Rachel Bartash, Emily Blumberg, Dana M Blyth, Saira Butt, Beata Casanas, Brian Chow, Lisa Clough, James B Cutrell, Ryan K Dare, Saumil Doshi, Jorgelina de Sanctis, David Dobrzynski, Jason Faulhaber, Christina Fiske, Christopher Graber, Aniruddha Hazra, Leonard B Johnson, Anna Kaltsas, Prathit A Kulkarni, Taesung Kwon, Allison Lastinger, Mikyung Lee, Anne-Marie Leuck, Paola Lichtenberger, Raul Macias Gil, Luis A Marcos, Caline Mattar, Brionna Matt, Eileen K Maziarz, Michael T Melia, Alfredo J Mena Lora, Benjamin Miko, Subhashis Mitra, Saman Nematollahi, Nwora Lance Okeke, Georgina Osorio, Frederico Perez, Varun K Phadke, Katya Prakash, Gail Reid, David J Riedel, Christian Rojas-Moreno, Natalie Mariam Salas, Kathryn Schlaffer, Mohammad Mahdee Sobhanie, Daniel A Solomon, Ann Stapleton, Judy Streit, George Thompson, Trevor C VanSchooneveld, Manasa Velagapudi, Holenarasipur R Vikram, Devin Weber, Scott Weisenberg, Darcy Wooten, Christa S Zerbe

**Affiliations:** Section on Infectious Diseases, Wake Forest University School of Medicine, Winston-Salem, North Carolina, USA; Division of Infectious Diseases, Massachusetts General Hospital, Harvard Medical School, Boston, Massachusetts, USA; Section of Infectious Diseases, Temple University Hospital, Lewis Katz School of Medicine, Philadelphia, Pennsylvania, USA; Division of Infectious Diseases, Rush University Medical Center, Chicago, Illinois, USA; Division of Infectious Diseases, Medstar Georgetown University Hospital, Georgetown University School of Medicine, Washington, District of Columbia, USA; Division of Infectious Diseases, University of Alabama at Birmingham, and Birmingham Veterans Affairs Medical Center, Birmingham, Alabama, USA; Division of Infectious Diseases, Mayo Clinic College of Medicine and Science, Rochester, Minnesota, USA; Division of Infectious Diseases, West Virginia University School of Medicine, Morgantown, West Virginia, USA; Infectious Diseases Society of America, Arlington, Virginia, USA; Division of Infectious Diseases, Southern Illinois University School of Medicine, Springfield, Illinois, USA; Division of Infectious Diseases, University of Connecticut School of Medicine, Farmington, Connecticut, USA

**Keywords:** acuity, clinical volume, infectious diseases fellowship, medical education

## Abstract

The Infectious Diseases Society of America Training Program Directors Committee met in October 2022 and discussed an observed increase in clinical volume and acuity on infectious diseases (ID) services, and its impact on fellow education. Committee members sought to develop specific goals and strategies related to improving training program culture, preserving quality education on inpatient consult services and in the clinic, and negotiating change at the annual IDWeek Training Program Director meeting. This paper outlines a presentation of ideas brought forth at the meeting and is meant to serve as a reference document for infectious diseases training program directors seeking guidance in this area.

## Teaser

Infectious Diseases (ID) Training Program Directors have observed an increase in clinical volume and acuity. Specific goals and strategies to manage the volume and acuity while preserving quality education in both the inpatient and ambulatory settings are described

The annual IDWeek Fellowship Training Program Director meeting allows program directors to exchange ideas to tangibly improve their programs. “High-Volume, High-Acuity, and High-Impact Learning: Tips and Tricks for ID” was selected as the theme in 2022. The Program Directors Committee members identified 4 subtopics via consensus: improving program culture, negotiating change, and maintaining educational priorities in outpatient and inpatient settings. Attendees heard presentations, engaged in discussion, then reported on consensus discussion points, common challenges, and specific actionable items. Summary meeting notes formed the foundation for this manuscript. While there are no perfect solutions, this article offers potential strategies for programs to consider.

## WHY IS PROGRAM CULTURE IMPORTANT? WHAT CAN PROGRAMS DO TO IMPROVE CULTURE?

The Accreditation Council for Graduate Medical Education (ACGME) recognizes the link between program culture and trainee well-being and stipulates that programs are responsible for trainee well-being by promoting self-care and maintaining a positive culture [1]. The culture within training programs encompasses values, attitudes, and behaviors that shape learning environments, with “sociable working environment” cultures preferred by trainees [2]. Potentially modifiable barriers to wellness and opportunities to improve culture include a lack of control over time; inconsistent, unfriendly, or unhealthy work environments; lack of wellness initiatives; and lack of space to recharge or reflect [3]. Positive work environments require investment from leadership, faculty, and trainees alike as inconsistencies between institutional messaging and practice can result in frustration [1]. Gestures of mutual respect are vital to program culture and can include allowing fellow input into on-call scheduling as well as advocating for improved workspaces. Trainee involvement in developing, evaluating, and assessing interventions is crucial, and those initiatives driven by trainees may be most effective [4]. Frequent feedback sessions with fellows and leadership on initiatives should be planned to guide continuous workplace improvement.

Healthy workplace culture requires attention to work-life integration, and programs can implement changes to positively influence this balance, including adjusting conference times to align with workflow, attending coverage of pager calls during dedicated educational time, and rotating presentation responsibilities among fellows and faculty. Programs should solicit trainee input on desired activities and consider hosting social events not tied to performative activities. Allowing fellows to dictate some conference topics may bolster ownership of learning opportunities. The following section and Table 1 offer specific suggestions regarding structural changes to support healthy workplace culture. Importantly, program culture can be sustained through external infectious diseases (ID) networks that enhance diversity, enthusiasm, and applicant pools. Programs can build networks by collaborating with others to host joint conferences, research symposia, mentorship opportunities, and interest groups.

**Table 1.** Strategies to Optimize Education While Managing High Clinical Volume and Acuity

	Inpatient Setting	Outpatient Setting
Personnel/team structure	Creation of nonteaching services to offload volume	Multidisciplinary (advanced practice clinicians, pharmacy and nursing staff, social workers) engagement and participation in fellows’ education
	Creation of mechanism for overflow service at peak volume loads	Protected time to supervise and teach (avoid scheduling meetings during clinic time)
	Alternative rounding to improve efficiency (table rounds, etc)	Prescreen fellows’ list of patients in clinic to improve efficiency and preidentify learning issues
	Fellows/faculty on service excused from outpatient duties	Provide brief teaching on relevant content
	Creation of follow-up service that absorbs patients with minimal ID decision needs who still require monitoring	Familiarize with scheduled patients before the clinic starts
	Creation of multidisciplinary OPAT teams that handle post-signoff medication adjustments and questions	Senior fellow autonomy (ie, incorporate graduated supervision strategies based on learner needs)
	Alternate timing of new consults between services to allow protected time from new consults (ie, one accepts new consults from 7 Am to 12 Pm, the other accepts new consults from 12 Pm to 5 Pm)	
Teaching strategies^a^	Develop chalk talks or mini-lectures to teach on common topics [5]	One-minute preceptor model that provides a framework for efficient delivery of real-time clinical pearls and “bite sized” education while also soliciting the learner's perspective [6]
	Signposting of clinical pearls and education	Self-directed case-based learning (eg, literature review, SNAPPS model) SNAPPS model: (1) summarize briefly the history and findings; (2) narrow the differential to 2 or 3 relevant possibilities; (3) analyze the differential by comparing and contrasting the possibilities; (4) probe the preceptor by asking questions about uncertainties, difficulties, or alternative approaches; (5) plan management for the patient's medical issues; and (6) select a case-related issue for self-directed learning [7]
	Attending supplied literature postrounding to supplement clinical discussions	Modify your teaching approach based upon time available [8]
	Attending or fellow provides one article per week for team for discussion	Incorporate high-yield outpatient topics in the core curriculum series
	Engaging other learners if available to provide short didactic sessions supplemented by attending input	Weekly or monthly outpatient clinic education meeting or case conferences
	Bingo cards or checklists for other learners to cover high-yield areas	Send an article then discuss in person or virtually, and provide email summary with article
	Engagement with social media, podcasts, and alternative educational content outside of rounds	Interactive questions (eg, utilize audience response system) for engagement during group teaching
	Use of strategies to improve clinical efficiency and teaching such as specified templates and use of technology in the physical rounding space (eg, screens or projectors) to enable easy access to materials	Provide medical literature of topics discussed and summary of case highlights at the end of the clinic day
	Teaching by modeling (eg, having learners observe how you counsel patients on medication adherence or negotiate removal of infected hardware with the attending surgeon) is an efficient way to couple teaching with patient care	Send a teaching pearl/take home points for one interesting case each day
	Clinically focused rounds in radiology or microbiology laboratory	Provide nonclinical teaching such as billing, coding, insurance issues, responding to patient and staff messages, communication skills to interprofessional teams and patients, and patient flow in clinic Use the EHR to evaluate provider-level patient outcomes
		Have learners write one of their learning goals for the clinic session on an index card at the beginning of clinic and one thing they learned during clinic on the other side of the card at the end of the clinic session [9]
Operational	Use of EHR for consult requests	Increase visit durations for patients seen by fellows
	Use of nurse/coordinator triage system for all calls/pages	Cap of number of patients and clinic slots
	Structured templates in notes regarding mechanism and timing of patient specific communication	Minimize last-minute add-ons or create nonteaching outpatient clinics for overflows and add-ons
	Routing antibiotic approval calls to dedicated stewardship personnel	Stagger clinic schedules (when multiple fellows are supervised by one staff)
	Medication adjustment questions for patients not being followed directed to OPAT or nonservice ID clinician	Block time dedicated to nondirect patient care activities (messages, clinic notes) and give protected catch-up time
	New consult time cutoff for nonurgent consults	Promote telemedicine and e-consult approach for uncomplicated cases to reduce workload of in-person visits
	Use of e-consults to decrease curbsides and external queries	Graded volume based on level of training
	Use of caps/thresholds for fellows/teaching teams	Protected time in clinic from hospital and other call responsibilities (have advanced practice clinicians or faculty hold pager for the duration of clinic)
	Guidelines for appropriate ID consultation with accessible tools for hospital teams	Consider reducing outpatient clinical duties when rotating on busy services
	Identification of stakeholders and building teams for quality improvement	Ensure adequate staff for clinical and pharmacy support including prior authorization and other paperwork
	Continued advocacy for academic support and appropriate reimbursements	

Abbreviations: EHR, electronic health record; ID, infectious diseases; OPAT, outpatient parenteral antimicrobial treatment; SNAPPS, summarize, narrow, analyze, probe, plan, and select.

^a^Teaching strategies may be appropriately applied to either the inpatient or outpatient setting and are not necessarily exclusive or universally applicable.

## WHAT STRATEGIES CAN BE USED TO MANAGE VOLUME AND INCREASE TIME FOR EDUCATION ON THE INPATIENT CONSULT SERVICES?

High patient volumes negatively impact education, and implementation of census limits or “caps” in internal medicine residency programs are associated with improved resident well-being, duty hours, and conference attendance [10, 11]. While uniform census caps exist for internal medicine residents, no such caps exist for ID fellows. As such, programs must determine best practices to manage inpatient consult volume with time for education. Table 1 details the methods that programs may consider to preserve the balance between volume and education.

Rounding efficiency and teaching time are impacted by tasks that interrupt learning. Specifically, the impact of pages, calls, and messages warrants attention. Programs may consider utilizing nurses, advanced practice clinicians, or other staff to triage and route consults and calls. Primary teams may communicate consult requests through the electronic health record (EHR) rather than paging. System-wide procedures that address consult timing (ensuring consults are placed before a specific time) allow for more predictable workflow and teaching time during rounds. Programs may consider diverting some inquiries to pharmacists or stewardship teams.

The structure of rounds influences the balance between volume and education. Additional consult services or nonteaching teams represent one method to decrease fellow workload. These services may be fixed or may operate as an overflow service.

Strategies to incorporate regular teaching even when there are high clinical volumes include teaching in the context of specific patients rather than a set curriculum. Posting teaching points, discussing one article or guideline per day/week, incorporating board review questions or microbiology rounds, and summarizing key points after rounds are also effective strategies. Alternative learning platforms, including social media, podcasts, and virtual learning modules, can be incorporated during or outside of rounds.

## WHAT AMBULATORY EXPERIENCES AND STRATEGIES CAN BE USED TO OPTIMIZE OUTPATIENT ID TRAINING?

Outpatient experiences and education are integral components of comprehensive ID training. Various ambulatory models exist and include weekly or biweekly clinics and ambulatory blocks that vary in duration. Dedicated outpatient rotations can provide focused experience in subspecialty ID clinics (transplant, hepatology, travel/tropical medicine, orthopedics, sexually transmitted infections, and outpatient parenteral antimicrobial treatment [OPAT]).

While participating in these clinics offers experiential learning, outpatient training should complement focused education, including case-based learning and didactics. Teaching content should include the evaluation and management of patients with known or suspected infections in ambulatory settings as well as strategies to optimally use the EHR, coordinate care in interprofessional healthcare teams, and communicate with patients and families.

High clinical volumes, pressure to maintain work relative value unit outputs, and lack of adequate support limit time for teaching. Logistical barriers may exist, such as commuting to ambulatory clinical sites and sharing resources (eg, computer terminals, examination rooms, and workspaces) with other healthcare team members. Addressing outpatient messages and clinical documentation while balancing education and other requirements can be challenging as well. Strategies to optimize outpatient education are delineated in Table 1.

## HOW SHOULD PROGRAM DIRECTORS APPROACH NEGOTIATING CHANGE WITH LEADERSHIP?

Negotiating support for substantive changes in ID fellowships should begin by assessing the needs of specific stakeholders: fellows, faculty, patients, divisions, and healthcare systems (Table 2). Programs should determine what support is needed and from whom. Such assessments require data on consult volume, workflow, and duty hours and metrics on patient safety, quality and satisfaction, and provider quality of life and burnout. Needs can be further classified as perceived, expressed, or relative to those of peer programs.

**Table 2.** Needs Assessment Worksheet

Possible Issues/Barriers in Fellowship	Specific Problem	Potential Solution
Resources	Lack of protected time to participate in didactic conferences because shift to virtual learning leads to multitasking at computer	Transition back to in-person didactic conferences. Set expectation that the attending answer the pager during didactic time
Professionalism	Fellows feel uncomfortable paging attendings overnight for consult questions	Set expectation that on-call attending contacts on-call fellow before 8 Pm or at end of the day with best way to be in touch overnight (pager vs phone)
Patient safety and teamwork	Fellows unaware of how to report safety issues	Incorporate safety report training during orientation
Faculty teaching and supervision	Faculty are less engaged with teaching because their time is uncompensated without reduction in their own clinical work	Advocate with division leadership ways to support and reward faculty engaged in teaching (eg, teaching awards, monetary compensation, protected time)
Evaluation	Minimal feedback provided from faculty to fellows after time on service together	Set expectations with faculty that over the lunch hour on the last day of service, attending and fellow have lunch together and provide 15 min for feedback session
Educational content	Minimal teaching on healthcare disparities in the core curriculum	Set expectation that every faculty speaker at a didactic teaching session incorporates how their topic is influenced by, or influences health disparities
Diversity and inclusion	Lack of engagement from fellows in DEI efforts	Develop a “fellowship arm” or representative to the DEI committee for the program
Clinical experience and education	Surpassing 80-h work week	Develop assessment of which services lead to work hour violations and revisit structure to identify how fellow time can be better supported
Fellow well-being	Fellows reporting high levels of burnout	Use listening sessions between fellows and program leadership to identify main stressors for fellows, and develop strategies to address these causes of burnout

Potential data sources: Accreditation Council for Graduate Medical Education survey, internal survey (institution-wide or unique to program), informal regional or peer program survey, Training Program Directors’ Committee, and list-serv.

Abbreviation: DEI, diversity, equity, and inclusion.

It is important to recognize that these needs may be shared nationally [12, 13]. Furthermore, fellows look to faculty for support; however, faculty themselves must be adequately supported [14]. Therefore, addressing the needs of the fellowship requires a partnership with faculty and leadership to advocate for better resources, compensation, and promotion including outside the training program setting [14–16].

If resources external to the division are required, advocacy lessons can be drawn from antimicrobial stewardship and OPAT. These programs promote opportunities for increased revenue and cost-savings (eg, shorter lengths of stay), while improving patient care and treatment outcomes [17–19]. Furthermore, leveraging partnerships with clinical services with high utilization rates to negotiate salary support may be effective. To effectively advocate for resources, clinical programs must demonstrate their impact on patient quality, safety, and financial metrics [20]. Aligning requests for resources with overall goals of the organization and executive leadership is essential.

## CONCLUSIONS AND FUTURE DIRECTIONS

Increased volume and acuity of clinical ID services have the potential to impact fellows’ education negatively. Implementing specific structural and cultural programmatic changes with support from leadership and stakeholders can mitigate these impacts, preserve an appropriate balance between clinical service and education, enhance fellow well-being, and potentially strengthen the ID workforce.

## Notes

***Acknowledgments.*** This work includes significant contributions made by the Infectious Diseases Training Program Directors Working Group: Emily Abdoler, Division of Infectious Diseases, University of Michigan Medical School; Kartikey Acharya, Division of Infectious Diseases, Medical College of Wisconsin; Marwan. M. Azar, Section of Infectious Diseases and Department of Laboratory Medicine, Department of Medicine, Yale School of Medicine; Rachel Bartash, Division of Infectious Diseases, Department of Medicine, Montefiore Medical Center/Albert Einstein College of Medicine; Emily Blumberg, Perelman School of Medicine at the University of Pennsylvania; Dana M. Blyth, Walter Reed National Military Medical Center; Saira Butt, Division of Infectious Diseases, Indiana University School of Medicine; Beata Casanas, Division of Infectious Disease, University of South Florida; Brian Chow, Division of Geographic Medicine and Infectious Diseases, Tufts Medical Center; Lisa Clough, Division of Infectious Diseases, University of Kansas School of Medicine; James B. Cutrell, Division of Infectious Diseases and Geographic Medicine, University of Texas Southwestern Medical School; Ryan K. Dare, Division of Infectious Diseases, University of Arkansas for Medical Sciences; Saumil Doshi, Medstar Washington Hospital Center; Jorgelina de Sanctis, Spectrum Health/Michigan State University; David Dobrzynski, Division of Infectious Diseases, University of Rochester Medical Center; Jason Faulhaber, Carilion Clinic; Christina Fiske, Division of Infectious Diseases, Vanderbilt University Medical Center; Christopher Graber, Veterans Affairs Greater Los Angeles Healthcare System and the David Geffen School of Medicine, University of California, Los Angeles; Aniruddha Hazra, Division of Infectious Diseases and Global Health, University of Chicago Medicine; Leonard B. Johnson, Division of Infectious Diseases, Ascension St John Hospital; Anna Kaltsas, Division of Infectious Diseases, Memorial Sloan Kettering Cancer Center; Prathit A. Kulkarni, Section of Infectious Diseases, Department of Medicine, Baylor College of Medicine; Taesung Kwon, Division of Infectious Diseases, Geisinger Medical Center; Allison Lastinger, Division of Infectious Diseases, West Virginia University; Mikyung Lee, Icahn School of Medicine at Mount Sinai, Mount Sinai Hospital; Anne-Marie Leuck, Division of Infectious Diseases and International Medicine, University of Minnesota; Paola Lichtenberger, Division of Infectious Disease, University of Miami Miller School of Medicine; Raul Macias Gil, Division of Infectious Diseases, Harbor-UCLA Medical Center and the David Geffen School of Medicine, University of California, Los Angeles; Luis A. Marcos, Stony Brook University; Caline Mattar, Infectious Diseases Fellowship, Washington University in St Louis; Brionna Matt, Section of Infectious Diseases, Temple University Hospital, Lewis Katz School of Medicine, Temple University; Eileen K. Maziarz, Division of Infectious Diseases, Duke University School of Medicine; Michael T. Melia, Johns Hopkins University; Alfredo J. Mena Lora, Division of Infectious Diseases, University of Illinois at Chicago; Benjamin Miko, Columbia University; Subhashis Mitra, Michigan State University; Saman Nematollahi, Department of Medicine, University of Arizona College of Medicine; Nwora Lance Okeke, Division of Infectious Diseases, Duke University School of Medicine; Georgina Osorio, Division of Infectious Diseases, Mount Sinai Beth Israel, Mount Sinai Morningside, and Mount Sinai West (Mount Sinai Health System, Icahn School of Medicine at Mount Sinai); Frederico Perez, University Hospitals Cleveland/Case Western Reserve University and Cleveland Veterans Affairs Medical Center; Varun K. Phadke, Emory University; Katya Prakash, Institute of Human Virology, University of Maryland School of Medicine; Gail Reid, Division of Infectious Diseases, Loyola University Medical Center and Edward Hines Jr Veterans Affairs Medical Center; David J. Riedel, University of Maryland; Christian Rojas-Moreno, Division of Infectious Diseases, University of Missouri–Columbia; Natalie Mariam Salas, Division of Infectious Diseases, Health Science Center, University of New Mexico; Kathryn Schlaffer, Institute of Human Virology, University of Maryland School of Medicine; Mohammad Mahdee Sobhanie, Division of Infectious Diseases, The Ohio State University; Daniel A. Solomon, Division of Infectious Diseases, Brigham and Women's Hospital, Harvard Medical School; Ann Stapleton, Eisenhower Health; Judy Streit, Iowa Carver College of Medicine; George Thompson, University of California, Davis Medical Center; Trevor C. VanSchooneveld, Division of Infectious Diseases, University of Nebraska Medical Center; Manasa Velagapudi, Creighton University School of Medicine; Holenarasipur R. Vikram, Mayo Clinic Arizona; Devin Weber, Thomas Jefferson University Hospitals; Scott Weisenberg, New York University Grossman School of Medicine; Darcy Wooten, University of California, San Diego; and Christa S. Zerbe, National Institutes of Health.

***Patient consent.*** This manuscript is a “Perspectives” article that does not include clinical data. The design of the work conforms to current US standards and is considered institutional review board exempt.
